# Supplementary material for: Investigation of HIV-1 Gag binding with RNAs and lipids using Atomic Force Microscopy
Source: PLoS One. 2020 Feb 3;15(2):e0228036. doi: 10.1371/journal.pone.0228036 (PMC6996966; doi:10.1371/journal.pone.0228036)
Supplement: S1 File — (DOCX) [file pone.0228036.s001.docx]

**Supporting Information**

Investigation of HIV-1 Gag binding with RNAs and Lipids

by Atomic Force Microscopy

**A AFM cantilever tip calibration**

Au nanospheres of 2nm diameter were used for the calibration of the AFM cantilever tips. As shown in panel A of Fig A, λ is the actual AFM cantilever tip size, L is the measured size of the calibration sample, and D is the height of the same calibration sample. α and β are the effective front and back angles of the tip which are related to the actual front angle FA and back angle BA of the tip FA through the tilt angle, θ, of the AFM probe holder (fluid cell if imaging in liquid) as shown in panel B of Fig. A.


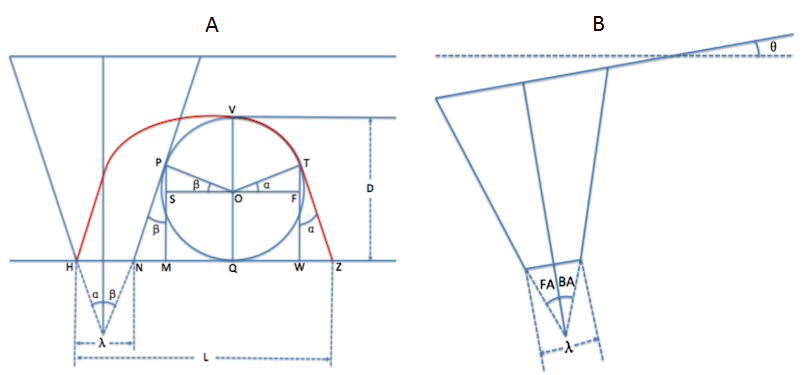


**Fig A AFM tip calibration**. (A) Schematic representation of the tip and a 2nm diameter Au calibration sphere. The red curve is the trajectory of the tip when it scans from left to right. (B) The configuration of the tip after it is placed into the AFM probe holder (fluid cell if imaging in liquid).

*α = FA + θ*

*β = BA - θ*

*HN = λ*

*OP =* $\frac{\boldsymbol{D}}{\boldsymbol{2}}$

*PS = OP sinβ*

*OS = OP cosβ*

*MQ = OS =* $\frac{\boldsymbol{D}}{\boldsymbol{2}}$ *cosβ*

*NM = PM tanβ = (PS + SM) tanβ =* $\frac{\boldsymbol{D}}{\boldsymbol{2}}$ *tanβ (sinβ + 1)*

*NQ = NM + MQ =* $\frac{\boldsymbol{D}}{\boldsymbol{2}}$ *tanβ (sinβ + 1) +* $\frac{\boldsymbol{D}}{\boldsymbol{2}}$ *cosβ =* $\frac{\boldsymbol{D}}{\boldsymbol{2}}$ *(secβ + tanβ)*

Similarly,

*QZ =* $\frac{\boldsymbol{D}}{\boldsymbol{2}}$ *(secα+ tanα)*

*L = HN + NQ + QZ = λ +* $\frac{\boldsymbol{D}}{\boldsymbol{2}}$ *(secα+ tanα + secβ + tanβ)*

For *θ = 10^0^*, *FA = BA = 20^0^*, then *α = 30^0^* and *β = 10^0^*, hence,

*L ≈ λ +1.46D*

Therefore, the actual tip size is

*λ = L – 1.46D*  (1)

And the effective tip size t for a sample with the height of D is

*t = L – D = λ + 0.46D*  (2)

**B Method for computation of the sample size**

The size of the sample is computed by a MATLAB script. First, a proper threshold value is selected to compute the height of the sample. The threshold value is so chosen to avoid the background noise. The height is the distance from the highest point of the sample to the threshold value cutoff plane. After the cutoff plane is set, the boundary of the sample can be obtained from the intersection of the sample and the cutoff plane. The length of the sample is defined as the distance between the two furthest points on the boundary. The width is defined as the distance between the two parallel lines restricting the object perpendicular to the direction of the length defined above.


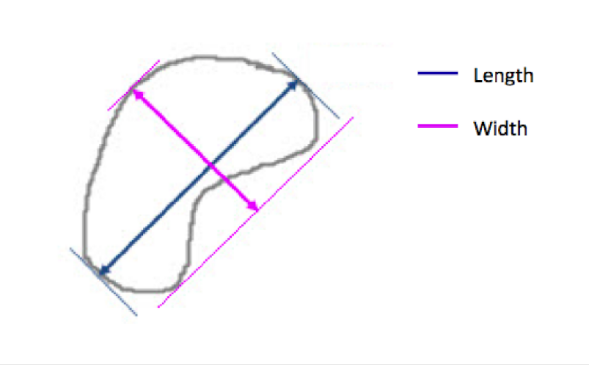


**Fig B Size measurement definition**. Schematic diagram of a sample with defined length and width.

**C Gel electrophoresis**

**Protocol for gel electrophoresis**

SDS-PAGE usually comprises of acrylamide, bisacrylamide, SDS, and a buffer with the proper pH. In the experiments reported here, tris-glycine SDS-PAGE was used with a 6% resolving gel and 5% stacking gel. The exact composition used in the Tris-Glycine SDS-PAGE is given in Table A. Here SDS is sodium dodecyl sulfate, APS is ammonium persulfate, and TEMED is N,N,N’,N’-tetramethylethylenediamine. In general, one SDS molecule is approximately bound to two amino acids regardless of the polypeptide sequence. Therefore, the migration of SDS bound proteins is proportional to the molecular weight of proteins. The procedure of SDS-PAGE gel electrophoresis used was as follows. First, 10mL 6% resolving gel and 4mL 5% stacking gel were prepared based on the composition for Tris-Glycine SDS-PAGE as given in Table A. APS and TEMED were added later for the 5% stacking gel. Then about 7mL of 6% resolving gel solution was added into the Mini-PROTEAN II Cell (BIO-RAD, Hercules, CA, USA). Next 1mL isopropanol was added on top to remove air bubbles at the surface of the resolving gel solution. After a wait of 20 minutes, the isopropanol was removed. Next, 40μL 10% APS and 4μL TEMED was added to the stacking gel solution. About 3mL of the 5% stacking gel solution was placed above the resolving gel solution. Finally a 10-well Mini-Protein comb (BIO-RAD, Hercules, CA, USA) was inserted on top of the stacking gel. The arrangement was allowed to form for 30 minutes before proceeding.

**Table A Tris-Glycine SDS-PAGE composition**


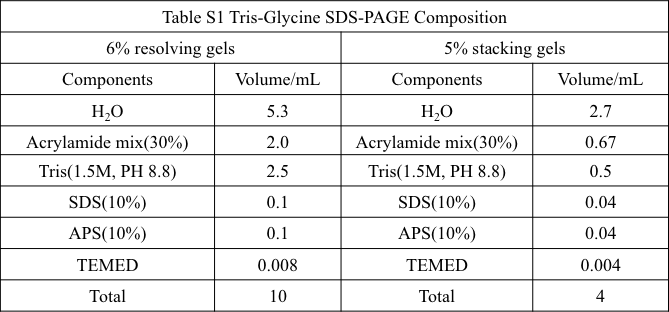


The sample solutions to be used were mixed with the dye bromophenol blue (Sigma-Aldrich, Merck KGaA, Darmstadt, Germany) to the desired concentration. Then the sample solutions were boiled for 5 minutes. Care was taken to seal all the sample solutions using parafilm. Next 1X SDS-PAGE loading buffer (25 mM Tris, 192 mM glycine, 0.1% SDS) was added into the gasket. The comb was gently removed. The dyed sample solutions were added into each comb slot carefully. Enough 1X SDS-PAGE loading buffer was added into the chamber that surrounded the gasket. It was confirmed that bubbles were generated at the bottom of the gel electrophoresis equipment. The following settings V = 120V, I = 100mA, T = 75minutes, and K in “Volts” mode were used. The gel run was started and continued until the color had almost reached the bottom. The equipment was disassembled to get the gel with the distinct bands located at the different positions. A plastic wedge plate was used to cut the top part of the gel. The gel was put into the transfer buffer (48 mM Tris, 39 mM glycine, 1.3mM SDS, 20%(v/v) methanol) using a clean container and then placed on a rotator for 20 minutes. A piece of Amersham Hybond 0.45μm PVDF blotting membrane (GE Healthcare Life Sciences, Pittsburgh, PA, USA) was cut to the same size as the gel plate. A corner was cut to distinguish the orientation. Two pieces of the blot paper were soaked in the transfer buffer for 20 minutes. The blotting membrane was soaked with pure methanol for 5 minutes. Next the methanol was removed and the transfer buffer was added and allowed to soak for 20 minutes. The blotting paper was placed on the semi-dry transfer cell. The blotting membrane was next positioned atop the blotting paper followed by the gel plate. A second blotting paper was placed on top of the gel. Air bubbles were removed by rolling a tube over the blotting paper. The following parameters V = 24V, I = 100mA, T = 28minutes, and K in “Volts” mode, were set to run the semi-dry transfer cell. Next 0.5g dry milk was added to 10mL 1X Tris-buffered saline (TBS, 50 mM Tris-Cl, pH 7.5 150 mM NaCl) buffer to get 5% milk TBS. The blotting membrane was put into a clean container, and 5% milk TBS buffer was added and incubated for 1 hour on a rocking platform in a freezer. The 5% milk TBS was then removed. Next 10mL reusable antibody (goat anti-HIV P24 in 5% milk TBS, 1:500) was added and incubated overnight on a rocking platform in a freezer. The blotting membrane was washed 3 times with 1X TBS buffer with 10 min intervals. Next 10mL 5% milk TBS was prepared with the addition of 5μL of another antibody (rabbit anti-goat secondary antibody). This was added into the blotting membrane and incubated for 90 minutes. The milk TBS was removed and the blotting membrane was washed 3 times with 1X TBS buffer. Enough developing solution (10mL 1M Tris-HCl(pH 9.5), 2mL 5M NaCl, 0.5mL 1M MgCl2, 33μL 50mg/mL NBT (nitro-blue tetrazolium chloride), and 16.5μL 50mg/mL BCIP (5-bromo-4-chloro-3'-indolyphosphate p-toluidine salt) was added to completely cover the blotting membrane. After the bands appeared, the blotting membrane was washed with 1X TAE (40mM Tris, 20mM acetic acid, and 1mM EDTA (Ethylenediaminetetraacetic acid)) buffer. The blotting membrane was dried with nitrogen gas. A picture of the blotting membrane was taken and then analyzed using the software ImagJ.

**Gel electrophoresis result**

The motivation for implementing gel electrophoresis measurement was to confirm the identity of the higher order multimer complexes found in the AFM measurements as corresponding to trimers or tetramers. The results from Tris-Glycine SDS-PAGE is shown in Fig C . The 50kDa protein marker indicates the bottom band corresponding to the 50kDa monomer. This is because the mass of the complete Gag monomer is about 55kDa and that of GagΔP6 is around 50kDa [8]. The middle band is just above the 75kDa calibration marker and closely aligns with the 100kDa calibration marker, corresponding to that of the Gag dimer as the mass of the GagΔP6 dimer should be 100kDa. The top band is in between the calibration mark for 150kDa and 250kDa, which means they are tetramers with approximate molecular weight of 200kDa for GagΔP6. For the Gag-ΨRNA complex the tetramer would have a mass around 236kDa if including one ΨRNA. These results confirmed that the complexes found in AFM measurements were tetramers rather than trimers. As shown in channels a and b in Fig C, the percentages of dimer and tetramers increased as the concentration of GagΔP6 increased from 0.5μM to 2μM. This result is consistent with the conclusion that the average radius of gyration *Rg* of GagΔP6 in solution measured by SANS is a monotonically increasing function of GagΔP6 concentration. This is reasonable because the size of the tetramer is greater than the size of the dimer, which in turn is again greater than the size of the monomer [57]. As shown in channels b and c or a and d of Fig C, the percentages of dimer and tetramers increased with the addition of ΨRNA for the same concentration of GagΔP6. This was also confirmed in the AFM measurements where ΨRNA can bind with GagΔP6 and facilitate GagΔP6 multimerization. As shown in channels c,f and g of Figs C, the percentages of dimer and tetramers increased even more when both ΨRNA and PI(4,5)P2 were added. Similar to ΨRNA, increasing PI(4,5)P2 lipid lead to increased GagΔP6 multimerization.


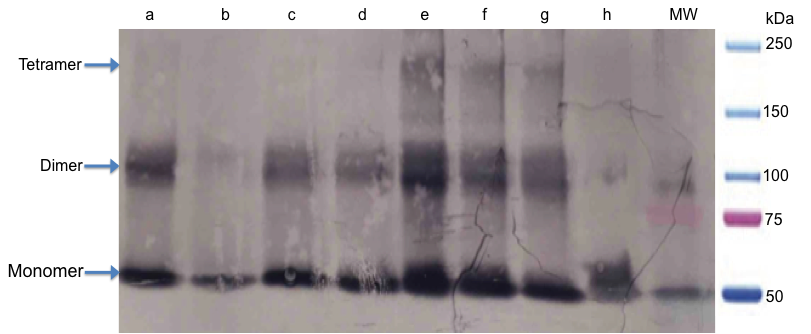


**Fig C Gel electrophoresis result** SDS-PAGE analysis (a) GagΔP6 (2μM). (b) GagΔP6 (0.5μM). (c) GagΔP6-ΨRNA (0.5μM : 0.5μM). (d) GagΔP6-ΨRNA (0.5μM : 2μM). (e) GagΔP6-ΨRNA (2μM : 0.5μM). (f) PI(4,5)P2-ΨRNA-GagΔP6 (0.5μM : 0.5μM : 0.5μM). (g) PI(4,5)P2-ΨRNA-GagΔP6 (2μM : 0.5μM : 0.5μM). (h) 50kDa Protein marker. (MW) Protein standards. The rightmost lane represents molecular weight of protein standards. Bands corresponding to monomer, dimer, and tetramer are indicated on the left. Note: The standard proteins bands beyond 100 kDa can be seen in the original gel but not in the Western blot since their large molecular weight resulted in inefficient transfer to the membrane.
