# Supplementary material for: Investigation of HIV-1 Gag binding with RNAs and lipids using Atomic Force Microscopy
Source: PLoS One. 2020 Feb 3;15(2):e0228036. doi: 10.1371/journal.pone.0228036 (PMC6996966; doi:10.1371/journal.pone.0228036)
Supplement: Attachment — Submitted filename: Response to Reviewers.docx [file pone.0228036.s002.docx]

Reply to Reviewers

Reply to Reviewer #1

***Changes made are highlighted in manuscript with track changes

Reviewer #1: The manuscript entitled “Investigation of HIV-1 Gag binding with RNAs and Lipids using Atomic Force Microscopy” by Chen et al, focused to investigate the molecular structure of complexes involving Gag protein with its multiple targets including -RNA, TAR polyA RNA and lipids PI(4,5)P2 by using Atomic force microscopy which provides direct visual information at the single molecule level. The size of individual components was also visualized and the presence of dimers, tetramers were observed in some cases. Although, the present study show some interesting indication of multimerization of GagP6 in presence of both PI(4,5)P2 and -RNA, the following concerns should be clarified before publication:

1. The major concern in the whole study is how the authors identify the complexes in their AFM images (Fig 7-10)? The images of RNA have distinct linear shape. As soon as the protein is added the whole complex become spherical or globular. At this stage, it becomes really difficult to assign whether the complex is really formed or not. The measurement of height, length, width is not enough to characterize the complex formation. This drawback poses concern on the conclusion drawn from the data set shown in Fig 7-10. To avoid this ambiguation, authors could make a construct where their specific RNA is attached at the end of double stranded DNA (~300 bp). In this construct, the advantage would be whenever the protein-RNA complex would be formed, it would show up as a feature containing a blob at the end of the dsDNA, where the RNA construct is situated. It would unambiguously show that the complex is formed, and further similar analysis could be done on those features. Another possibility to avoid this concern is to perform AFM time-lapse imaging, where the RNA would be imaged first and then different proteins, lipids and their combination could be added to visualize the complex formation in situ. Unless these issues are clarified, it is difficult to comment on the conclusions.

Answer: We thank the reviewer for the careful reading and analysis of the manuscript. We agree with the reviewer that size change alone is not sufficient to confirm binding. However, the goal in this manuscript was to study the change in the population distribution and structure of Gag with the addition of RNA and the lipid PIP2, not just whether RNA binding alone has happened. In addition to the size changes in the structure of Gag with RNA addition, we measure significant changes in the Gag monomer, dimer, and tetramer population. The change in the Gag multimer population with the addition of RNA (and also lipid) that was measured with the AFM was also confirmed through gel electrophoresis. We welcome the suggestion to confirm the binding of the RNA to Gag with a unique construct of dsDNA attached to the RNA, though there is a concern that this would disturb the Gag multimerization, which is the focus of our study. The second suggestion to do time-lapse imaging is also welcome and can be tried. But we note that in this proposal, as the Gag/RNA complex is lying on the substrate, the mobility of the RNA might affect the binding to the Gag that is later introduced. To avoid these complications in the experiments discussed here the RNA-Gag interactions (and those with lipids) were done in solution before the mixture is introduced into the AFM. We do not observe any changes to the complex during the AFM imaging consistent with the low mobility of the Gag/RNA complex.

2. Authors should elaborate on why the width of the -RNA becomes double in the dimer. As the length of the RNA is increased the width value should remain the same.

Answer: Please note that the definition of the width is the largest value perpendicular to the length, which is the most extended end to end distance. The width in the dimer being larger than the width of the monomer is probably due to overlap of the two RNA’s on dimerization. The width at this overlap region will necessarily be larger. A review of literature shows that the probable overlap of RNA monomers is through the SL1 head group which contains the palindromic sequence DIS [30-32]. Please see Fig. 1. This is consistent with the length of the dimer being less than twice the length of the monomer. We have now added modified sentences to page 9 para 2 line 12 to clarify this as follows: “This is probably due to overlap of the two RNA’s on dimerization. This assumption is reasonable based on the results from Refs. [30-32] and the potential overlap region is the palindromic DIS base pair region located in the SL1 loop of ΨRNA as shown in Fig.1(b).”

3. Authors mentioned that the GagP6 is cylindrical in shape, whereas in AFM image they appeared spherical. The reason behind that should be clarified. They have provided the model in Fig. 6, but enough experimental evidence is missing.

In Ref [2], the extended Gag is thought to have a cylindrical shape with a length of 20-30 nm and a diameter of 2-3 nm. However, in solution, based on Ref. [3-5, 57], Gag is known to be folded with the MA domain in close proximity to the NC domain. Our AFM measurement showed the height of the Gag is indeed 2 nm consistent with that expected for extended Gag molecule. For the Gag monomer, we measure the length to be 10.3 nm while the width is 6.2 nm. As mentioned above, in Ref. [3-5, 57], the Gag was observed to be folded over, with its N-terminal MA domain near its C terminal NC domain in three-dimensional space. The conjectured model in Fig. 6 is consistent with that of the folded Gag monomer expected in solution from Ref [3-5, 57] and the AFM size measurements. The conjectured model in Fig. 6 is also consistent with that of a fully extended Gag having a rod-like shape of 2 nm radius and 20-30 nm length postulated in the literature [2]. To further clarify this in the manuscript we have added the following modified sentences to page 11, para 3, line 4: “In neutron scattering and hydrodynamic experiments [4] the Gag was observed to be folded over, with its N-terminal MA domain near its C- terminal NC domain in three-dimensional space. The conjectured model in Fig. 6 is consistent with the measurements of the folded Gag monomer expected in solution and the AFM size measurements. The conjectured model in Fig. 6 is also consistent with that of a fully extended Gag having a rod-like shape of 2 nm radius and 20-30 nm length postulated in the literature [2].”

4. How did the authors calculate the width for elongated complexes? Did they measure at three different regions and then average it?

Answer: The procedure is described in the supplementary section. In the analysis, the width of a sample is defined as the largest distance perpendicular to the direction of the length. The length of the sample is defined as the distance between the two furthest points on the boundary of the sample. We developed a software program to do the height, length and width measurements for the 100’s of samples analyzed in each set of data. The length and width values are averaged for the many samples measured.

5. Did the authors separate the excess lipids from the complexes? If free lipid were present lipid patches should be observed on the surface in Fig. 9.

Answer: We did not observe lipid patches in these experiments. This is probably because of the low concentration of the lipids used and because they were premixed with Gag of the same low 0.5 μM concentration resulting in their binding and thereby leaving very little free lipids to form lipid patches.

6. Authors should compress the introduction section. The elaborate details of the Gag structure is not necessary rather the rationale of the present work should be mentioned clearly.

Answer: We appreciate the reviewer’s suggestion to compress the introduction. Following the suggestion, we have deleted information in the introduction which is not central to the measurements reported. The deleted lines are indicated in the manuscript with track changes.

We thank the reviewer for the questions and suggestions which have improved the presentation in the manuscript.

Reviewer #2:

Reply to Reviewer #2

***Changes made are highlighted in manuscript with track changes

This is the review of the manuscript entitled “Investigation of HIV-1 Gag binding with RNAs and Lipids using Atomic Force Microscopy” by Chen et al. In this manuscript the authors used solution AFM experiments to probe the multimerization and conformations of Gag in the presence of PIP2 and RNA. The authors show good resolution of the AFM using 2nm gold particles and go on to resolve ΨRNA as well as the TARpolyA RNA molecules absorbed to mica surfaces. They show a significant fraction of dimer ΨRNA absorbed on the mica and detect the U shaped conformation of Gag. I have found the study to be sufficiently rigorous and well presented, however there are important shortfalls that need to be addressed before the paper is accepted for publication.

Answer: We thank the reviewer for a careful reading of the manuscript and the conclusion that the manuscript is “sufficiently rigorous and well presented.” The answers to the questions raised by the referee are provided below.

Major Issues.

1- It is not clear how quantification of dimer Ψ RNA was performed? Based on the definition of the width measurements presented in the supplementary materials, the dimers should have much larger widths than 4 nm.

Answer: Regarding the quantification, the three dimensional smooth histogram of the length and width population distributions are fit to normal distributions to identify their respective mean values. The population distributions and the corresponding fits for Ψ RNA are shown in Fig. 3. The monomer distribution has a mean length of 17.9 ± 0.2nm, a mean width 1.01 ± 0.02nm and a mean height of 1.10 ± 0.01nm. The ΨRNA dimer distribution has a mean length of 34.6 ± 0.3nm, mean width of 3.8 ± 0.1nm and a mean height of 1.10 ± 0.01nm. The width of the dimer is larger than the height due to the overlap of the two Ψ RNA molecules at the junction. Based on literature the probable overlap of RNA monomers is through the SL1 head group which contains the palindromic sequence DIS [30-32]. The following new sentences are also provided on page 9 para 2 line 12 of the manuscript. “This is probably due to some overlap of the two RNA’s on dimerization. This conclusion is reasonable based on the results from Refs. [30-32] and the potential overlap region is the palindromic DIS base pair region located in the SL1 loop of ΨRNA as shown in Fig.1(b).”

2- When GagΔP6 is observed on mica, the histograms show Gag molecules with a diameter of ~6 nm and length of 10 nm, the dimers show diameter of 6nm and length of 20 nm. Analyzing the AFM image however, It didn’t look like the identified dimers are longer, instead they were showing a larger overall diameter. This was also true for the tetramers, the authors should explain this discrepancy

Answer: The GagΔP6 size population distributions for the 858 samples observed on mica are shown in Fig. 5 (b) and 5 (c). We did not analyze the images in terms of a “diameter” as the images were far from circular. We analyze only the length and the width. As described (additional details in the supplementary section), the definition of the length is as the longest end to end distance in the image above the noise threshold. The width is the largest value of the image perpendicular to the length. From the analysis of the population distribution in Fig. 5(b), the first peak with a mean length of 10.3 ± 0.1nm and width of 6.2 ± 0.1nm corresponds to the GagΔP6 monomer. The second peak with a mean length of 20.0 ± 0.2nm and width of 6.2 ± 0.1nm corresponds to the GagΔP6 dimer. The third peak with a mean length of 29.0 ± 0.3nm and width of 12.9 ± 0.2nm corresponds to the GagΔP6 tetramer. A potential conformation for the GagΔP6 is given in Fig. 6 based on the size observed here and from literature. In solution, based on hydrodynamics and neutron scattering data [3-5, 57], Gag is known to be folded with the MA domain in close proximity to the NC domain giving rise to the flattened C-type conformation conjectured in Fig. 6. For the case of the dimer: Based on the AFM measured dimensions of a length of 20.0 nm and width of 6.2 nm (same as monomer) a model of the GagΔP6 dimer would have two monomers connecting back to back through their CA-CA interaction [4]. Note that the CA-CA interaction between Gag molecules is well documented [4]. The corresponding modified sentences are on page 11 para 3 line 9 as:

“For the case of the Gag dimer, based on the AFM measured dimensions of a length of 20.0 nm and width of 6.2 nm (same as monomer) a model of the GagΔP6 dimer would have two “C” shaped monomers connected back to back through their CA-CA domain interaction [4]. The CA-CA domain interaction leading to dimerization has been reported in the literature [4].”

3- The authors are assuming that what they observed is a stoichiometric representation of the ingredients, however their method only shows topological features with no specificity. To be more specific, when the authors say they show PIP2-GagΔP6-RNA complexes, there is no independent verification that the blub they are looking at actually contains all those components. If the authors can provide independent verification of complexes it will significantly strengthen the study, otherwise the authors should rewrite and de-emphasize the stoichiometry of their complexes. In short, the way the paper now reads, it implies independent verification, where there is none.

Answer: The gel electrophoresis data presented in supplementary section for the GagΔP6, RNA and PIP2 lipid combinations provide complementary confirmation for the different complexes that are present in the same solution mixtures analyzed with the AFM. Note also that the size and population distribution are distinct and show changes for the complexes on the addition the RNA and PIP2 to the GagΔP6 solution. Please also note that the goal of our study is the “morphology of Gag, ΨRNA, and their binding complexes with lipids” with the AFM.

To address the reviewers concern the following addition has been made to the end of the conclusion: “The gel electrophoresis data presented in supplementary section for the GagΔP6, ΨRNA and PI(4,5)P2 lipid combinations provide complementary confirmation for the different complexes that are present in the same solution mixtures analyzed with the AFM. The size and population distribution measured for the various complexes are distinct and show changes for the complexes on the addition the ΨRNA and PI(4,5)P2 to the GagΔP6 solution.”

4- Basic mutagenesis of Gag would have helped the study become a lot more interesting, for example the G2A mutant?

Answer: We appreciate the suggestion by the referee to use additional mutagens of Gag such as the G2A mutant. With the current manuscript we hope to demonstrate that such AFM based morphological studies of Gag complexes adds value and is complementary to other methods. In future we hope to use mutant Gag to further probe Gag self assembly pathways and kinetics.

5- There needs to be a significant discussion about what the mica surfaces are doing in to skew the selection of molecules from solution, also Gag molecules typically interact with lipid bilayers, how is that effecting the experimental results has to be addressed.

Answer: This study was limited to probing the morphological changes in Gag that were known to occur with the addition of RNA and the lipid PIP2 using the AFM. Freshly cleaved mica surfaces are atomically smooth and clean and therefore ideal for imaging at an Angstrom scale. When imaging the various complexes formed, we always mixed the complex in the appropriate molar ratio and then the solution was incubated for 3 hours in order to confirm that the different distributions of the complexes have reached equilibrium before the solution was introduced to the AFM fluid cell containing the mica substrate. In addition, the same freshly cleaved mica substrate was used for the investigating all the Gag complexes: Gag, Gag/RNA, Gag/PIP2 and Gag/RNA/PIP2. Thus it is valid to make comparison of the changes in the morphologies and population statistics of monomers, dimers and tetramers with the addition of RNA or PIP2 to Gag in solution. During imaging on the mica substrate, no dynamical evolution of the complexes were observed. Following the reviewer’s suggestion we have clarified this further on page 6, para 1 line 7 as:

“When imaging the various complexes GagΔP6 formed, we always mixed the components in the appropriate molar ratio and then incubated the solution for 3 hours in order to confirm that the different distributions of the complexes have reached equilibrium before the solution was introduced to the AFM fluid cell containing the mica substrate. In addition, the same freshly cleaved mica substrate was used for the investigating all the Gag complexes: GagΔP6, GagΔP6/RNA, GagΔP6/ PI(4,5)P2 and GagΔP6/RNA/ PI(4,5)P2. Thus it is valid to make a comparison of the changes in the morphologies or population statistics of monomers, dimers and tetramers with the addition of RNA or PI(4,5)P2 to GagΔP6 in solution. During imaging on the mica substrate, no dynamical evolutions of the complexes were observed.”

Regarding the Gag molecule interaction with lipid bilayers: We agree with the reviewer that this is an important interaction in the formation of the HIV virus. In this manuscript we limited ourselves to just the Gag morphology changes with RNA and the lipid PIP2 as they are thought to precede the interaction of the Gag/RNA/PIP2 multimer complex with the lipid bilayer. We plan future studies in the direction suggested by the reviewer.

We again thank the reviewer for the careful reading of the manuscript, thoughtful questions and suggestions for future studies. The clarifications requested have improved the manuscript.
